# Mechanoimmunology: Are inflammatory epigenetic states of macrophages tuned by biophysical factors?

**DOI:** 10.1063/5.0087699

**Published:** 2022-08-29

**Authors:** Nikhil Jain, Janet M. Lord, Viola Vogel

**Affiliations:** 1 Institute of Inflammation and Ageing, University of Birmingham, Birmingham, United Kingdom; 2 School of Chemical Engineering, University of Birmingham, Birmingham, United Kingdom; 3 MRC-Versus Arthritis Centre for Musculoskeletal Ageing Research, University of Birmingham, Birmingham, United Kingdom; 4 Department of Health Sciences and Technology, Institute of Translational Medicine, ETH Zurich, Zurich, Switzerland

## Abstract

Many inflammatory diseases that are responsible for a majority of deaths are still uncurable, in part as the underpinning pathomechanisms and how to combat them is still poorly understood. Tissue-resident macrophages play pivotal roles in the maintenance of tissue homeostasis, but if they gradually convert to proinflammatory phenotypes, or if blood-born proinflammatory macrophages persist long-term after activation, they contribute to chronic inflammation and fibrosis. While biochemical factors and how they regulate the inflammatory transcriptional response of macrophages have been at the forefront of research to identify targets for therapeutic interventions, evidence is increasing that physical factors also tune the macrophage phenotype. Recently, several mechanisms have emerged as to how physical factors impact the mechanobiology of macrophages, from the nuclear translocation of transcription factors to epigenetic modifications, perhaps even DNA methylation. Insight into the mechanobiology of macrophages and associated epigenetic modifications will deliver novel therapeutic options going forward, particularly in the context of increased inflammation with advancing age and age-related diseases. We review here how biophysical factors can co-regulate pro-inflammatory gene expression and epigenetic modifications and identify knowledge gaps that require urgent attention if this therapeutic potential is to be realized.

## I. INTRODUCTION

Despite the significant progress made in medicine, we still struggle today with treating many long term, progressive diseases that are typically accompanied by chronic inflammatory processes. As the clinical focus historically has been on organ-specific diseases, medicine was compartmentalized along organ-specific divisions, which presents challenges to recognizing generic pathogenic mechanisms. Even though organ-specific cell niches have distinct biochemical and biophysical characteristics, inflammatory processes are at the core of many chronic diseases and common pathomechanisms are now emerging. The importance of understanding the role of biomechanical signals in the inflammatory status of cells, including macrophages and fibroblasts, is exemplified by the impact of inflammation in human ageing and age-related disease. Advancing age is accompanied by an increase in systemic inflammation, termed inflammageing,^1^ which predisposes to several age-related diseases with an inflammatory component, including cardiovascular disease, non-healing post injury, Alzheimer's disease, and cancer.^2^

The term “inflammation” refers to a cascade of events that often starts with an infectious challenge or a sterile injury that activates immune cells, notably macrophages, to produce a range of soluble mediators (cytokines) that mediate the immune system response. Eventually, this inflammatory response is actively terminated to ensure homeostasis and the healing of tissue, i.e., “resolution of inflammation.”^3^ Lipopolysaccharides (LPS) are frequently used as immune cell activators for *in vitro* cell culture experiments, as they mimic the interaction of immune cells, including macrophages, with the cell wall of gram-negative bacteria. Macrophages, the major cellular drivers of the inflammatory process, originate from two distinct lineages:^4,5^ tissue resident macrophages are seeded into organ tissues during early embryonic development and self-maintain locally throughout adult life with minimal contribution from circulating monocytes, whereas blood monocyte-derived macrophages home to sites of injury or infection to respond to the challenge. These are short-lived cells and originate from adult hematopoietic stem cells. Macrophages play pivotal roles in the maintenance of tissue homeostasis achieved through their differentiation into two broad phenotypes, M1 pro-inflammatory macrophages and M2 macrophages, which have a more anti-inflammatory function. While there is a broad spectrum of intermediate states between M1 and M2,^6,7^ the graded balance of these main phenotypes influences the chronicity and outcomes of the inflammatory response.^7^ For example, if macrophages persist in a pro-inflammatory M1 phenotype, they can contribute to chronic inflammation and fibrosis;^8,9^ in contrast through their functions in the clearance of apoptotic cells and cellular debris and secretion of anti-inflammatory cytokines, the M2 macrophages contribute to the resolution of inflammation to reestablish tissue homeostasis.^10^ In addition to macrophages that display the M1 or M2 phenotype, also a variety of organ-specific subsets exists.^11^

**FIG. 1.**
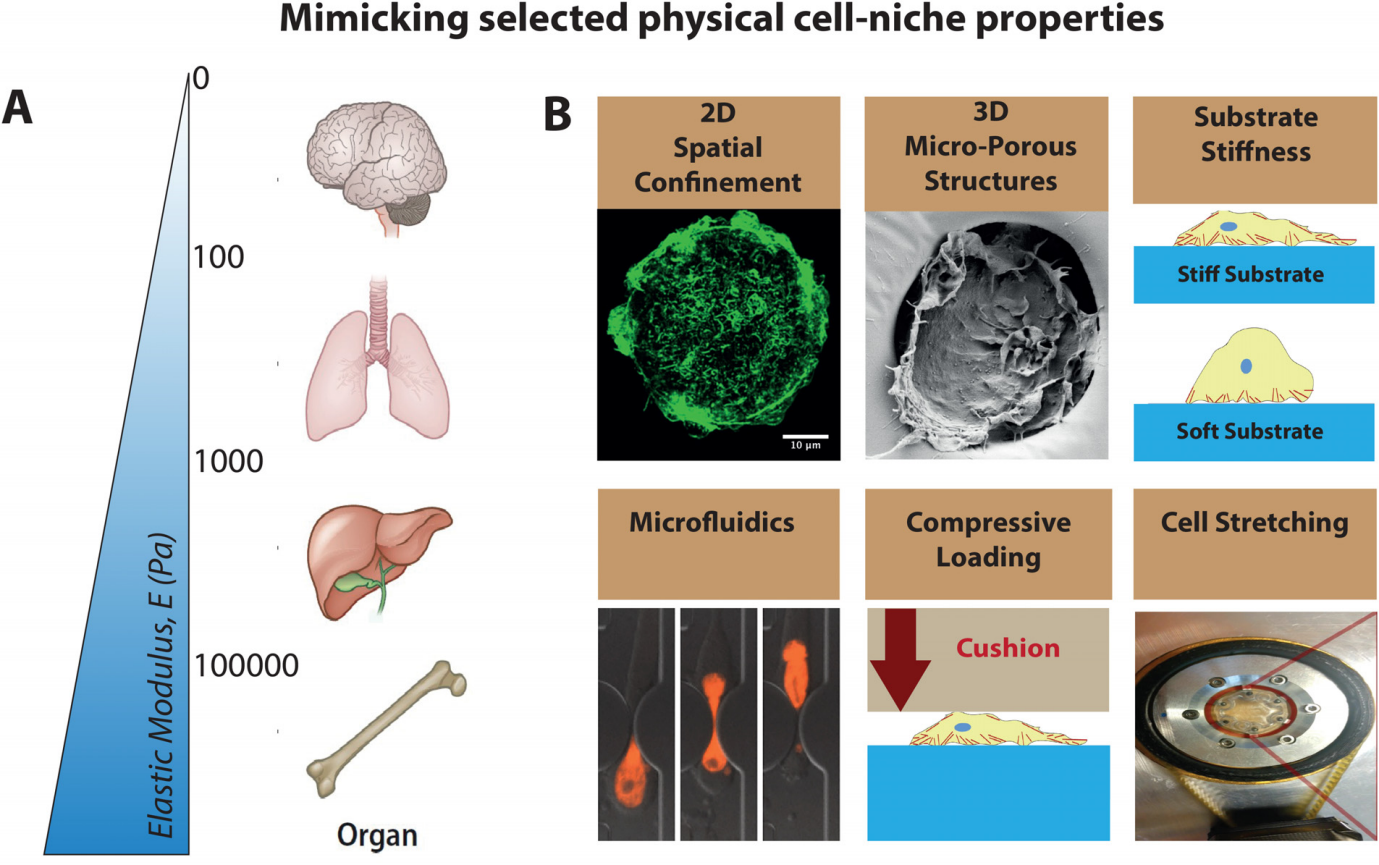
Multifactorial tissue specific physical properties and selected bioengineering tools to elicit their respective roles on regulating cell signaling and function. (a) The stiffness is different for different organs. Organ cartoons reproduced with permission from Jain *et al.*, Annu. Rev. Biomed. Eng. **21**, 267–297 (2019). Copyright 2019 Annual Reviews, Inc. (b) Microfabricated cell niches to probe cell behavior under controlled conditions to recreate selected physical properties of cell-niches. Cartoons adapted and modified from Refs. 12, 33, 46, and 74. Reproduced with permission from N. Jain and V. Vogel, Nat. Mater. **17**, 1134–1144 (2018). Copyright 2018 Springer Nature Limited. Reproduced with permission from Elacqua *et al.*, PLoS One **13**, e0195664 (2018). Copyright 2018 Authors, licensed under a Creative Commons Attribution (CC BY) license. Reproduced with permission from N. J. Walters and E. Gentleman, Acta Biomater. **11**, 3–16 (2015). Copyright 2015 Authors, licensed under a Creative Commons Attribution (CC BY) license.

While niche-specific transcriptomic and epigenetic profiles as well as secretome are well documented for multiple tissues and various physiological contexts, cell phenotypes are also determined by a range of biophysical factors and stimuli.^12–20^ Micro- and nanofabricated materials and devices have allowed researchers to probe how cell behavior and function depend on the physical properties of their microenvironment, including flow, stiffness of the microenvironment, extracellular matrix (ECM) tethering to the substrate, the ECM viscoelastic properties, topography, and spatial confinement as well as tensile or compressive forces^12–46^ [Fig. 1(a)]. The role of the tissue microenvironment is well recognized in mechanobiology and is particularly well studied for mesenchymal cells, as physical cell-cell communication and between cells and their extracellular niche environments are also crucial to direct cell fate.^15,20–28,30,32,34,36,39–42,47–51^ Cells sense mechanical properties of their environment by pulling on it or pushing against it. Much progress has been made in the molecular understanding of how mechanical stimuli are sensed and transduced by mesenchymal cells into biochemical signals (mechanotransduction), which then regulates gene transcription processes and subsequently cell phenotype.^30,32,34,36,39–42^

In contrast, current research in immune cell biology is still dominated by a cell centric view, rather than taking a more holistic view that considers how cells react to both biochemical and biophysical microenvironmental factors. With respect to immune cells, T cell activation is affected by substrate stiffness^37,43^ and nanoporous substrates,^29^ and macrophage phenotype is greatly impacted by a multitude of different physical factors^14,33^ (as discussed in detail below in Secs. II–V). Far less is known about the mechanobiology of other immune cells, including B cells^38,52^ and dendritic cells.^31,35^ What has been revealed is that various mechanotransduction-induced signaling pathways trigger the translocation of transcription factors from the cytoplasm to the nucleus,^53–56^ including myocardin related transcription factor-A (MRTF-A), which is released from actin upon stress fiber assembly and yes-associated protein (YAP)/TAZ whose dephosphorylation is induced in response to the opening of mechanosensitive ion channels, including piezo channels.^57–63^ Beyond regulating the transcription of genes, nuclear translocation of transcription factors also triggers various epigenetic modifications as reviewed further below.

Despite this rapid emergence of mechanobiology and the study of underpinning mechanisms, knowledge gaps exist as to how these mechanisms relate to chronic inflammatory diseases and, in particular, the relevance to inflammatory macrophages. How to intervene to reestablish homeostasis after infection, injury or pathological tissue transformation is still one of the biggest challenges of regenerative medicine. As the field of immunology has only recently started to appreciate the impact of physical factors on macrophage phenotype regulation, a review of the literature is provided here to summarize what is known of how biophysical factors can regulate inflammatory gene expression and macrophage polarization in the context of inflammation and disease, and what research questions require urgent attention. Current evidence highlights already how the changing landscape of biomechanical signals during inflammation directs several inflammatory biochemical intermediates in response to pro-inflammatory activation, which then converge onto transcriptional and epigenetic modifications (histone modifications and DNA methylation) and changes in the chromosome landscape as reviewed here. As such these changes could be either modulated by altering the biophysical properties of the microenvironment or be targeted by specific drugs modulating mechanosensitive signals. Since these nuclear events and epigenetic modifications have previously been shown in other cell types to be dependent on the nucleoskeleton or nuclear meshwork, the data taken together also suggest the existence of an unknown and yet to be explored role of the nuclear meshwork in driving inflammatory activation of macrophages, which requires urgent attention. Even though recent reviews have individually provided a glimpse onto these regulatory events,^14,33,64^ a comprehensive review is missing in the field.

## II. BIOCHEMICAL AND BIOPHYSICAL CHARACTERISTICS GIVE CELL NICHES THEIR ORGAN-SPECIFIC IDENTITY

Organ-specific cell niches are complex and comprise many cell types, all in close proximity to each other. Each organ is characterized by organ-specific cell types that are supported and surrounded by stromal cells common to most organs, including fibroblasts as well as immune cells that have sentinel functions in sensing injury and infection. This latter group includes primarily tissue resident macrophages and macrophages derived from activated, homed-in circulating macrophages. Other common cellular tissue residents can include adipocytes as active endocrine producers, as well as perivascular mesenchymal stem and endothelial cells. These cell societies communicate with each other biochemically and physically. They respond to global metabolite composition^65^ as well as to localized autocrine and paracrine signaling to which all cell types contribute. Most importantly in the context of this review, macrophages can sense physical cues in their environment, including soft or more rigid substrates^66–70^ or microstructured environments,^12,13,71,72^ or cyclic strain,^73^ and respond in a mechanosensitive manner [Fig. 1(b)].

Cell niches are not only characterized by the resident cells but also have characteristic ECM compositions, assembled into complex meshworks of nanofibrils that are undergoing constant remodeling by the synergistic actions of various cell types. Some of these cells synthesize the ECM or its components, while they themselves or others contribute by secreting cross-linking and proteolytic enzymes.^75,76^ Although most of the complex organ-specific niches serve highly specialized functions, inflammatory processes or pathologies of different organ types are likely based on common principles and shared mechanisms. As the complex secretome of macrophages is shifting in response to external stimuli, the intercellular niche communication is tuned bidirectionally. The secretome of macrophages exposed to calcium oxalate crystals, for example, is shifted toward proteins involved mainly in “inflammatory response” and “fibroblast activation” and activates the expression of alpha Smooth Muscle Actin (a-SMA) in renal fibroblasts.^77^ Vice versa, the secretome of preconditioned mesenchymal stem cells drives polarization and reprogramming of M2a macrophages toward an interleukin (IL)-10-producing phenotype, thus improving their regenerative and immunomodulatory capacities^78^ and in cancer. The tumor microenvironment is rich in crosslinked collagen^79^ and other ECM components, creating niches for tumor-promoting macrophages.^80,81^ This bidirectional communication can also explain pharmacological side effects or be exploited therapeutically. Naïve, M1, and M2 macrophages are affected by the antipsychotic drug Olanzapine, known to cause metabolic side effects by promoting obesity and diabetes, as the macrophage-derived secretome is sufficient to confer olanzapine-mediated insulin resistance in human adipocytes.^82^ Macrophages thereby play key roles in shaping healthy cell niches, as well as the tumor microenvironment, tumor immunity, and the response to immunotherapy.^4,83^ Fibrotic pathologies^84,85^ and ageing^86,87^ are also associated with major changes in the ECM composition and crosslinking, which alters the physical properties of the cell niches, to which the cells respond in a negative feedback loop. This ultimately leads to a loss of organ function. Indeed, the “big five” contributors to fibrotic pathologies of a range of organs have been proposed to include: macrophages, myofibroblasts, matrix, mechanics, and miscommunication.^88^ Yet, how the synergy of physical and biochemical stimuli together orchestrate cell niche functions is not fully understood. This is particularly striking when we consider that the failure of proinflammatory macrophages to revert to anti-inflammatory macrophages is associated with the onset of fibrotic diseases.^88,89^

## III. CELL MORPHOLOGY AS A PHYSICAL PHENOTYPE STABILIZER OR MARKER OF MACROPHAGE ACTIVATION

**FIG. 2.**
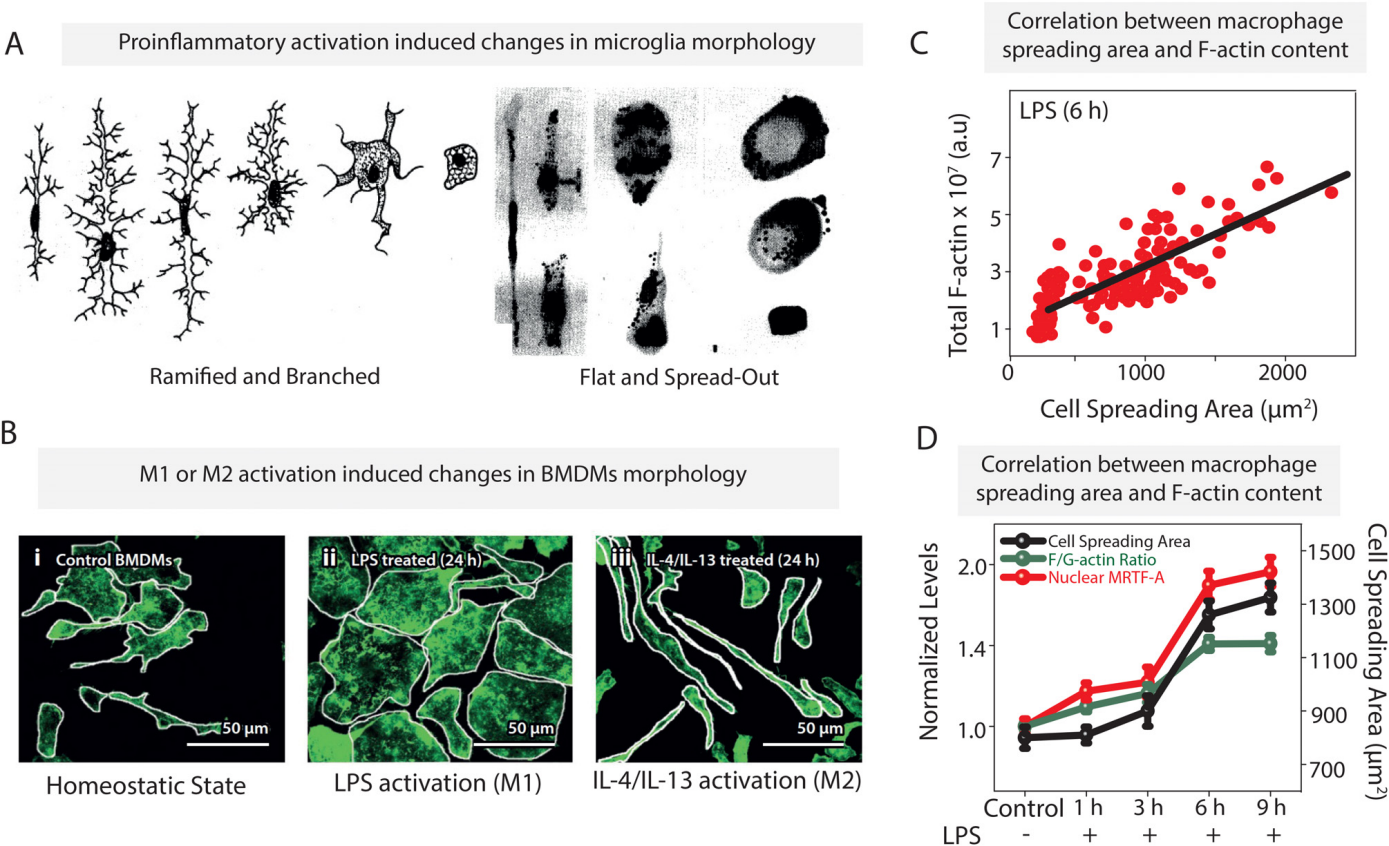
Macrophage activation leads to changes in cellular morphology, actin polymerization, and nuclear translocation of transcription factors: (a) early hand-drawn sketches of unactivated microglia (left) and activated microglia (right) show characteristic ramified and flat morphology, respectively (permissions are not needed).^90^ (b) Representative images of (i) control (M0), (ii) LPS-treated (M1), and (iii) IL-4/IL-13-treated (M2) bone marrow derived macrophages (BMDMs) stained for F-actin (green).^12^ (c) Cell spreading area vs total F-actin content in LPS-treated BMDMs.^12^ (d) Time course changes in cell spreading areas, nuclear levels of MRTF-A and F/G-actin ratios in LPS-treated BMDMs for different periods of time.^12^ Data adapted and modified from Ref. 12. Reproduced with permission from N. Jain and V. Vogel, Nat. Mater. **17**, 1134–1144 (2018). Copyright 2018 Springer Nature Limited.

More than a century ago, it was first reported that brain-resident macrophages, i.e., microglia, have a characteristic morphology defined by a small cell body with fine ramified processes [Fig. 2(a), left].^90^ The first hand-drawn images of resting and activated microglia were sketched by Merzbacher in 1909, which interestingly suggested significant changes in microglia morphology upon inflammatory activation.^90^ The LPS-activated microglia were found to be rounder and flatter with a pancake-like morphology [Fig. 2(a), right].^90^ These hand-drawn sketches also suggested an increased spreading area upon pro-inflammatory activation with LPS. In the absence of bioengineering tools and techniques at that time it was not possible to deduce any potential regulatory and functional link between changes in microglia morphology and their inflammatory state. It took the scientific community more than a century to finally establish these links.

As for tissue resident macrophages, also the pro-inflammatory activation of bone marrow derived macrophages (BMDMs) with LPS leads to major morphological changes: with a delay of a few hours, the cell spreading area increases significantly and the macrophages transition from an elongated to a more rounded cell shape [Fig. 2(b)],^12^ which is concomitant with enhanced actin polymerization [Fig. 2(c)]. Interestingly, removal of LPS restores the resting morphology, although over a longer timescale, further confirming a functional link between macrophage morphology and pro-inflammatory phenotype.^12^ In contrast, pro-healing, anti-inflammatory differentiation of macrophages to an M2 phenotype, using interleukin (IL)-4/IL-13, results in elongated morphologies^13^ [Fig. 2(b)]. Elongation factor, defined as the length of the longest axis divided by the length of the shortest axis across the cell nucleus, increases more than threefold in M2 macrophages.

## IV. PROINFLAMMATORY AND PRO-HEALING MACROPHAGES RESPOND DIFFERENTLY TO SPATIAL MICRO- AND NANOSCALE FEATURES

With the advent of bioengineering tools and techniques, we can now address in detail the key question: Does macrophage activation first lead to a change of gene expression profile which is then followed by a change in cell morphology, or vice versa? Recent data show that certain cell morphologies, as imposed by spatial constraints, can help in stabilizing macrophage phenotype. To ask whether staging a fully fledged inflammatory response requires actin polymerization, bioengineered tools were used to spatially confine macrophages on micropatterned adhesive islands of defined sizes and shapes. For example, the production of inflammatory cytokines including IL-6 and tumor necrosis factor-alpha (TNF-α) was greatly reduced when the spreading of BMDMs was restricted by culturing them on adhesive islands of appropriate size, in hemispherical pores, or in a close-packed cell layer.^12^ This shows nicely that imposing spatial confinement can indeed modulate the pro-inflammatory response of macrophages. Importantly, using fibronectin coated stripes or nanofibers that can cause cell elongation, it has been shown that BMDMs can be polarized toward the M2 phenotype without any additional stimulation with IL-4/IL-13.^13,82,83^ Furthermore, by confining macrophages in 2D and 3D substrates, our lab has shown that the expression of pro-inflammatory secondary response genes (*IL-6*, *iNOS*, *CXCL9*), necessary for prolonging the process of inflammation, is dependent on macrophage spreading.^12^ Moreover, these spatial constraints can dampen or synergize with chemical stimulants to decrease or increase the inflammatory phenotype of macrophages. These data suggest that morphological cues may be used to complement or dampen the effects of cytokines or other pro-inflammatory factors already present in the cell microenvironment.

These findings are significant, as tissue resident macrophages are found in a range of tissue-specific sizes and shapes, surrounded by niches with tissue-specific characteristics including stiffness, ECM composition, fiber density, and fiber tension.^91^ While M1 macrophages greatly increase in circularity and cell area upon activation *in vitro*,^12^ M2 macrophages elongate with little change in the cell spreading area.^12,13^ Such changes may have relevance to disease pathogenesis, for example, macrophage elongation is also seen in certain disease states such as atherosclerosis.^92,93^ In addition to topographical cues, physiological levels of interstitial flow are also able to stabilize the M2-like phenotype.^94^ Also substrate stiffness tunes M2 activation, whereby cells on softer substrates show enhanced M2-like phenotypes as regulated by RhoGTPase signaling.^68^ While many other effects have described how physical factors impact macrophage behavior and phenotype,^14,33,95,96^ our emphasis here is to ask how selected physical factors impact the cytokine gene transcription machinery of macrophages. For example, what is the impact of environmentally imposed elongation on the M1 phenotype? One report suggested that enforcing cell elongation has only a limited effect on the M1 response,^12^ whereas another report suggests that elongation protects macrophages from M1 polarization by inflammatory stimuli.^13^ These differential findings clearly suggest the need for further research.

## V. MACROPHAGE MORPHOLOGICAL CHANGES ARE DRIVEN BY THE REMODELING OF THE ACTIN CYTOSKELETON

Changes in cellular morphology are coupled with a reorganization of the cytoskeleton. As the first fragile contacts, i.e., focal adhesions, become mechanically reinforced, assembly of a mature cytoskeleton progresses rapidly, guided by the spatial locations of the point of contact to the extracellular matrix. Major insights into these processes were gained via research on mesenchymal cells through the micro- and nanofabrication of adhesive contacts. These studies illustrated how the cytoskeletal filaments are bundled along with the primary directions of force transmission through the cell, as imposed by the spatial presentation of environmental anchor points.^42,97–99^ Microcontact printing of adhesive islands revealed that changes in fibroblast cell morphology are associated with the reorganization of the cytoskeletal architecture.^100,101^ For example, a triangular fibroblast shows triangular pockets of polymerized F-actin around the nucleus whereas a circular cell shows circular rings of F-actin around the nucleus.^100^ However, establishing such connections in macrophages is more difficult as macrophages do not have actomyosin fibers. The majority of studies have largely focused on quantifying the levels of F-actin and myosin-II contractility during macrophage activation [Fig. 2(c)]. An increase in the cell spreading area during pro-inflammatory activation of macrophages is coupled with a significant increase in actin polymerization and formation of actin microfilaments (F-actin) within 3–6 h post-LPS treatment [Fig. 2(c)].^12^ Monomeric actin (G-actin) constitutes about 60% of cellular actin prior to LPS stimulation, which increases the F/G actin ratio and, thus, shifts the balance toward actin polymerization [Fig. 2(c)].^12^ These increased polymerized actin levels go along with increases of the cell size; thus, spatial confinement of macrophages restricts their spreading and reduces actin polymerization.^12^

Initiating actin polymerization involves the phosphorylation of paxillin on tyrosine 118 and Neural Wiskott-Aldrich syndrome protein (N-WASP) on serine 484/485, two actin-regulatory proteins important for actin polymerization and reorganization.^102^ Importantly, an increase in actin-polymerization is necessary to elicit the pro-inflammatory response of macrophages. The depolymerization of actin, using pharmacological inhibitors like latrunculin-A, results in a significant decrease in the expression of pro-inflammatory genes and cytokines and is also coupled with a significant decrease in the cell spreading area.^12^ Similar experiments using cytochalasin-D and other drugs targeting actin polymerization and pathways have established that the actin cytoskeleton is a key mediator in the process of macrophage proinflammatory activation.^103–105^ Given the mechanosensitive nature of actin and the importance of actin polymerization in regulating the staged response of macrophages, it should be noted that the levels of p-myosin light chain upon LPS stimulation, i.e., a measure of cell contractility, remained unchanged during macrophage pro-inflammatory activation.^12^ Even though the total cellular contractility was found to be similar, traction force microscopy (TFM) suggested higher traction forces in M1 macrophages upon LPS/interferon (IFN) γ stimulation, as compared to M0 mouse BMDMs.^106^ It should be noted that in human BMDMs, an opposite effect was found using TFM, i.e., that M1 macrophages generate significantly less force than M0 or M2 macrophages,^107^ suggesting that a careful and detailed analysis of macrophage contractility as a function of stimulants and substrate properties needs to be performed. Also, the details of how mechanical forces and spatial cues reinforce and reorganize the cytoskeleton in macrophages have still not been fully characterized.

## VI. THE RELEASE OF ASSOCIATED TRANSCRIPTION FACTORS DUE TO ACTIN POLYMERIZATION UPREGULATES THE PRO-INFLAMMATORY RESPONSE

**FIG. 3.**
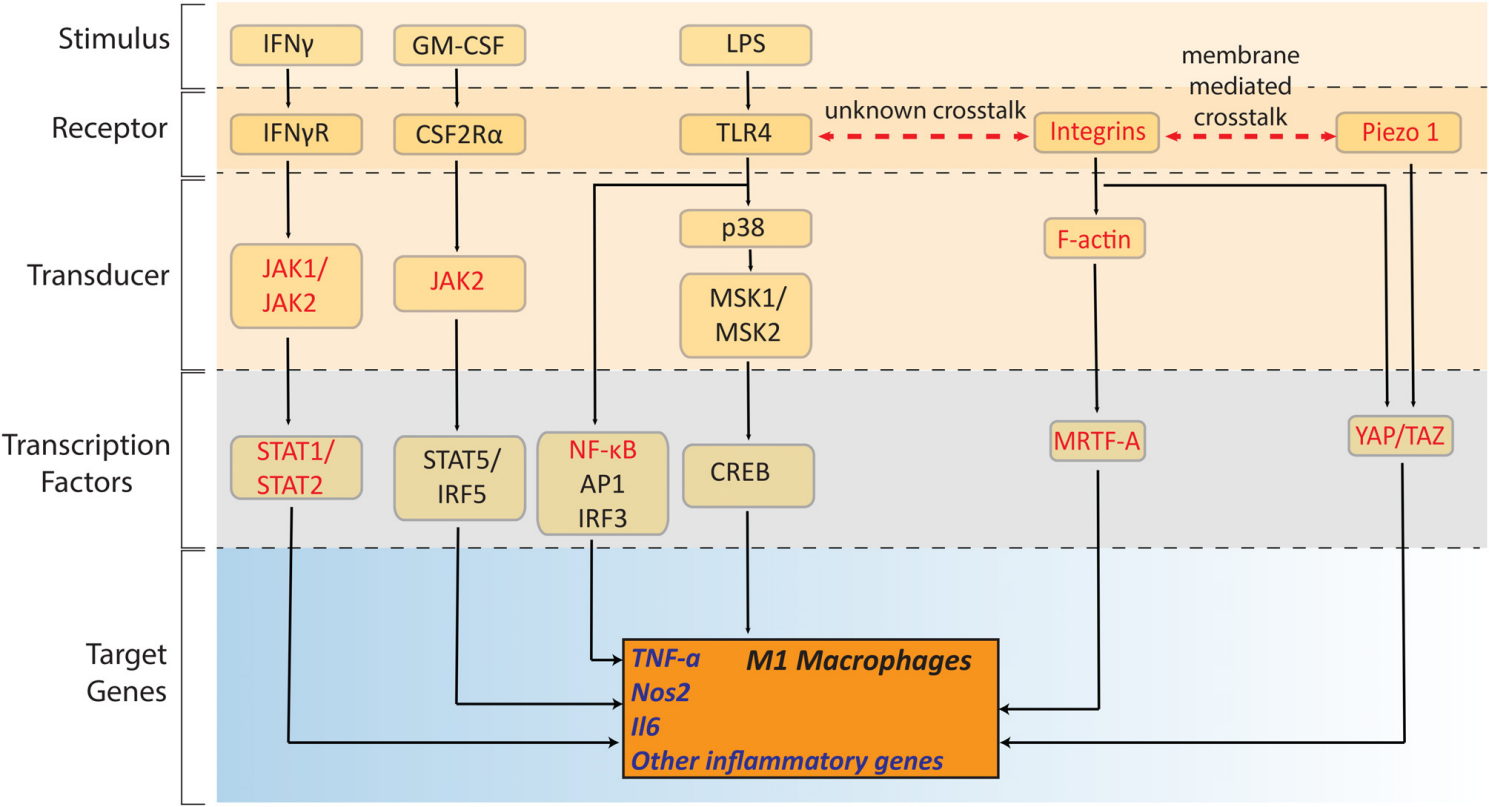
Mechano-regulation of the pro-inflammatory transcription in macrophages: Chemical and metabolic signaling pathways implicated in M1 macrophage polarization. An initial stimulus leads to the activation and nuclear translocation of sequence-specific transcription factors that eventually mediate changes in the transcriptional output. Note that conventional signaling diagrams do not consider the possibility that some of the signaling processes are mechanoregulated. Various signaling steps were recently described to be regulated by cellular sensing of physical factors which we marked here in red. This includes recently uncovered mechanosensitive transcription factors like MRTF-A and YAP, and how they are under the regulation of integrins and piezo channels. Abbreviations—CSF: colony-stimulating factor; GM-CSF: granulocyte macrophage colony-stimulating factor; IFN: interferon; IL: interleukin; IRF: interferon-regulatory factor; JAK: Janus kinase; MSK: mitogen- and stress-activated kinase; NF-κB: nuclear factor κB; Nos2: nitric oxide synthase 2; STAT: signal transducer and activator of transcription; TLR4: toll-like receptor 4; MRTF-A: myocardin related transcription factor-A; YAP: yes-associated protein 1. Cartoon adapted and modified from.^111^ Permission obtained from T. Lawrence and G. Natoli, Nat. Rev. Immunol. **11**, 750–761 (2011). Copyright 2011 Springer Nature Limited.

Actin polymerization has been shown to drive macrophage pro-inflammatory activation by regulating the nuclear-to-cytoplasmic shuttling of critical transcription factors.^12,108^ The transcription factor MRTF-A binds to G-actin in the cytoplasm, and the onset of actin polymerization leads to its release and results in its nuclear translocation. Upon complexation with the serum response factor (SRF), the complex drives the expression of various genes,^109^ yet the role of the MRTF-A-SRF complex has only recently been probed during macrophage activation^12,110^ (Fig. 3). Using MRTF-A knockout (KO) or SRF-KO BMDMs revealed that the MRTF-A-SRF complex positively drives the expression of critical pro-inflammatory genes like *IL-6* and *Nos2* (Ref. 12) (Fig. 3). Mechanistically, the regulation is mediated via recruitment of the nuclear factor κB (NF-κB), another critical transcription factor^110^ in the promoter region of pro-inflammatory genes, which requires MRTF-A. Another, central mechano-sensitive transcription factor complex, yes-associated protein (YAP), has recently been shown to promote the pro-inflammatory response by increasing *IL6* expression (Fig. 3), while concomitantly decreasing pro-healing, anti-inflammatory responses by decreasing arginase-I expression.^108^ LPS activation of macrophages leads to a higher accumulation of YAP in the nucleus, which depends on actin polymerization, and depolymerization of actin inhibits nuclear translocation of YAP, thereby reducing the secretion of pro-inflammatory cytokines like TNF-α^108^ (Fig. 3). Again, this influence was independent of myosin-II phosphorylation. Vice versa, genetic deletion of *YAP/TAZ* leads to impaired pro-inflammatory activation of macrophages. It is also important to note that unlike in fibroblasts, nuclear translocation of NF-κB is not sensitive to changes in macrophage spreading, suggesting cell-specific mechanosensitivity of certain classes of transcription factors.^12,100^

While the activity of most of these transcription factors during pro-inflammatory activation was characterized downstream of toll-like receptor 4 (TLR4) signaling, the major receptor that mediates LPS signaling,^111^ there is growing evidence that other surface molecules,^112^ mainly integrins^113^ and ion-channels like piezo-1,^114^ co-regulate the activity of these mechanosensitive transcription factors like MRTF-A and YAP (Fig. 3). As the piezo channel opening is regulated by membrane tension, which will change as cells bind and pull on their environment. It is expected that blocking integrin signaling decreases LPS induced production of TNF-α as well.^113^ Piezo1 activity promotes Interferon-γ and LPS-induced inflammatory and suppresses IL-4 and IL-13-induced healing responses,^114^ illustrating membrane-mediated crosstalk between these different membrane receptors, i.e., TLR4, integrins, and ion-channels.^113,115^ How the crosstalk between these receptors and transcription factors are regulated by the physical properties of macrophage niches in different tissue environments is unclear (Fig. 3), and understanding this will help in probing whether the physical properties of these niches tune macrophage differentiation and stabilize tissue-resident macrophage phenotypes.

Contrary to pro-inflammatory macrophage activation, the organization and levels of actin polymerization during pro-healing M2 macrophage activation are still not well characterized. Even though certain reports suggest that inhibiting actin polymerization and myosin contractility significantly reduce M2 activation, without altering their cell elongation,^13^ the specific role of the cytoskeleton during M2 activation needs further clarification.

## VII. MACROPHAGE POLARIZATION RESHAPES THE NUCLEAR ARCHITECTURE, NUCLEUS-CYTOSKELETON CONNECTIONS, INTERNUCLEAR GENOMIC SPACE, AND EPIGENETIC REGULATION

**FIG. 4.**
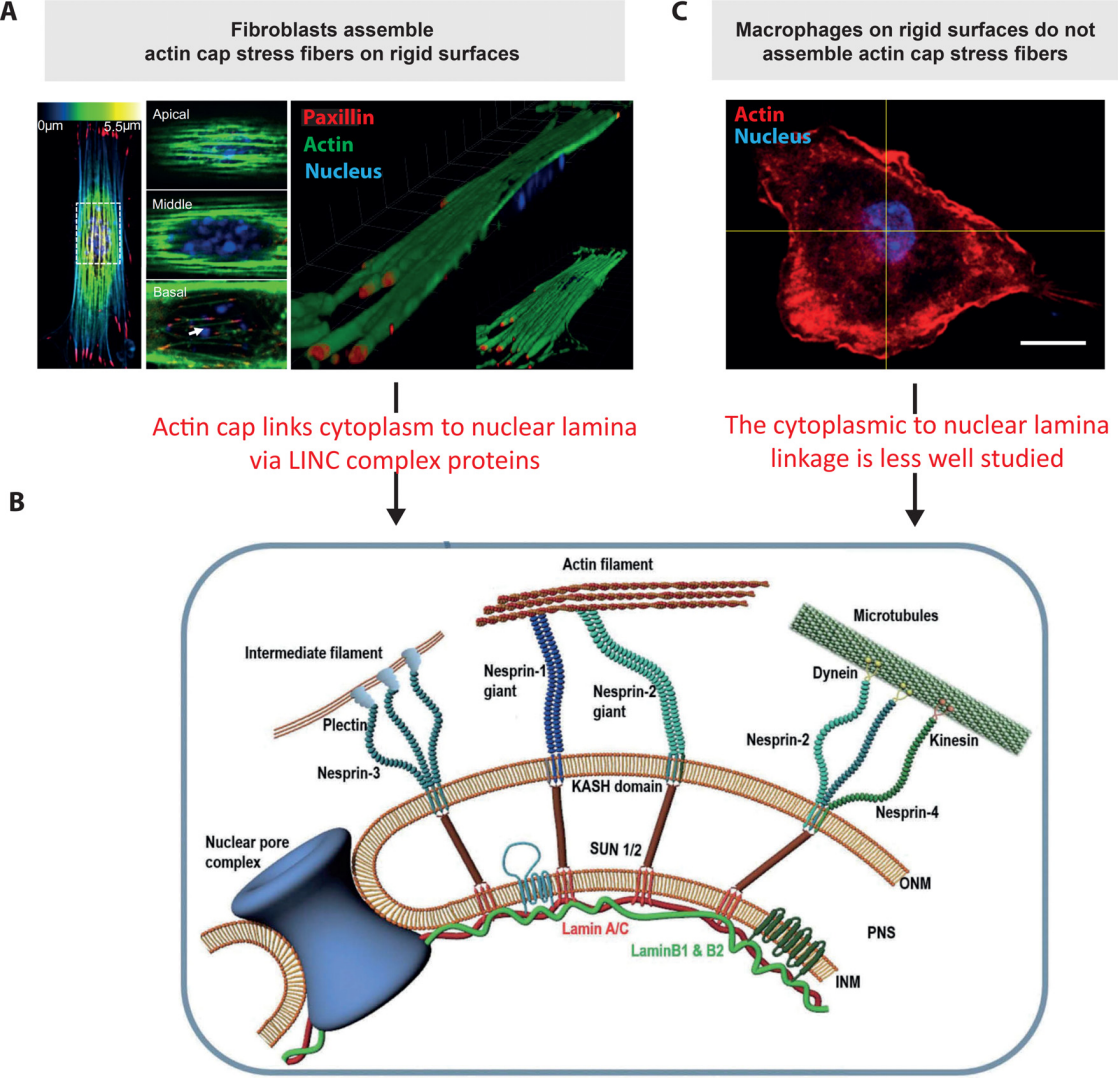
Physical coupling of the actin cap and the cytoskeleton to the nuclear lamina via the LINC complex proteins: (a) color coded height map of actin. Blue, green, and yellow colors represent the actin structure at the basal, middle, and apical plane (paxillin in red). Middle panel: Zoom in view of actin at apical, middle, and basal plane. Right panel: 3D reconstruction of actin and nucleus. Cartoon adapted and modified from.^118^ Reprinted from Li *et al.*, Biomaterials **35**, 961–969 (2014). Copyright 2014 Elsevier. (b) LINC complexes consist of KASH-domain-containing nesprin isoforms on the outer nuclear membrane (ONM) that are connected to the cytoplasmic actin filaments, intermediate filaments, and microtubules. SUN proteins interact with the nuclear lamina decorating the inner nuclear membrane (INM). SUN proteins and nesprins are connected through KASH-SUN interactions in the perinuclear space. Cartoon adapted and modified.^120^ Reproduced with permission from Sneider *et al.*, Cell Adhes. Migr. **13**, 50–62 (2019). Copyright 2019 Authors, licensed under a Creative Commons Attribution (CC BY) license. (c) Representative image of a single BMDM, stained for F-actin and nuclei, showing the absence of a conventional actin-cap.^12^ Scale bars, 10 *μ*m. Physical connections between nucleus and cytoplasm via LINC complex are not characterized in macrophages. Cartoons adapted and modified from.^12^ Reproduced with permission from N. Jain and V. Vogel, Nat. Mater. **17**, 1134–1144 (2018). Copyright 2018 Springer Nature Limited.

As cytoskeletal components mechanically couple the cell adhesions of the plasma membrane to the cell nucleus, the nucleus can get mechanically strained leading or at least contributing to its remodeling. In fibroblast and epithelial cells, highly tensed actin stress fibers, called the actin-cap,^116–118^ span the cell nucleus and compress it,^99,117^ which has a profound impact on the architecture of the nuclear lamina^119^ and the nucleus^117^ [Fig. 4(a)]. This is largely mediated via force transmission from extracellular anchor points via the cytoskeletal filaments, including intermediate filaments, to the nucleus, where Linker of Nucleoskeleton and Cytoskeleton (LINC) complexes couple the cytoskeletal filaments to the nuclear lamina [Fig. 4(b)].^120^ These LINC proteins establish a direct physical linkage from the cytoskeleton via the nuclear lamina to chromatin and, thus, play a pivotal role in nuclear mechanosensing [Fig. 4(b)].^120,121^ The inner nuclear membrane is thereby linked to the nuclear lamina via a meshwork of type V intermediate filaments, the nuclear lamins, and associated proteins.^122^ The nuclear lamina interacts with chromatin and tethers heterochromatin to the nuclear periphery. The term “heterochromatin” was coined by Emil Heitz to distinguish regions that remained strongly stained throughout the cell cycle from those that became invisible during interphase.^123^ Heterochromatin is associated with a dense chromatin structure, where genes are thought to be inaccessible to transcriptional factors necessary for gene activation. The main mammalian lamins are lamin-A and C (also called lamin-A/C) and the evolutionarily older lamins B1 and B2.^122^ Cryo-electron tomography insights into the molecular arrangement of the nuclear lamina reveal a fiber-like morphology of lamin networks decorated with globules, forming filaments that are packaged into a 14 nm thick layer (the lamina).^124–126^ Disruptions of either the LINC complex or its physical coupling to the nuclear lamina or of lamin self-assembly into filaments by point mutations directly impinge on the nuclear architecture leading to multiple disease conditions collectively known as laminopathies.^127^

In contrast to mesenchymal cells, blood born cells often must crawl through tiny constrictions to reach their destiny, including transmigration through the endothelium, which requires that they undergo extreme cellular and nuclear deformations.^128–130^ Even though the functional and regulatory role of the LINC complex has been extensively studied for mesenchymal cells and more recently for immune cells, their potential role in regulating macrophage homeostasis vs their inflammatory or pro-healing responses are still largely unknown [Fig. 4(c)]. As the assembly of conventional actin stress fibers has so far not been described for macrophages, how they establish a physical connection between cytoskeleton and nuclear lamina is not known. As the nuclear architecture and the nuclear lamina regulates gene expression by controlling the three-dimensional organization of genes and their distal regulatory sequences,^131–140^ the intra-nuclear space and chromosome rearrangement during macrophage differentiation, polarization, and inflammatory activation has yet to be revealed.

## VIII. MACROPHAGE POLARIZATION INDUCES CHANGES IN NUCLEAR MORPHOLOGY AND IN THE PHOSPHORYLATION OF MECHANO-SENSITIVE NUCLEAR ENVELOPE PROTEINS

As cell size and nuclear size are positively correlated, it has been shown that pro-inflammatory M1 activation of macrophages results in a significant increase in the nuclear projection area and nuclear volume.^12^ In contrast, during M2 activation, macrophage elongation correlates with an increased nuclear aspect ratio.^12^ LPS induced increases in nuclear projection area and volume are dependent on the level of polymerization of actin.^12^ Depolymerization of actin in LPS activated BMDMs results in a significant decrease in nuclear size.^12^ Lamin-A/C is abundantly expressed in most differentiated cells, but the amount of lamin-A/C varies greatly between immune cell types with macrophages and dendritic cells (another early initiator of inflammation) expressing high levels, but resting T and B cells expressing low to barely detectable amounts.^141–144^ Research on the role of lamin-A/C during inflammation has largely been focused on T cells, which show a significant upregulation of lamin-A/C upon activation.^145^ Lamin-A expression accelerates T cell receptor (TCR) clustering and leads to enhanced downstream signaling, including extracellular signal-regulated kinase 1/2 (ERK1/2) signaling as well as increased target gene expression contributing to T-cell activation.^144^

**FIG. 5.**
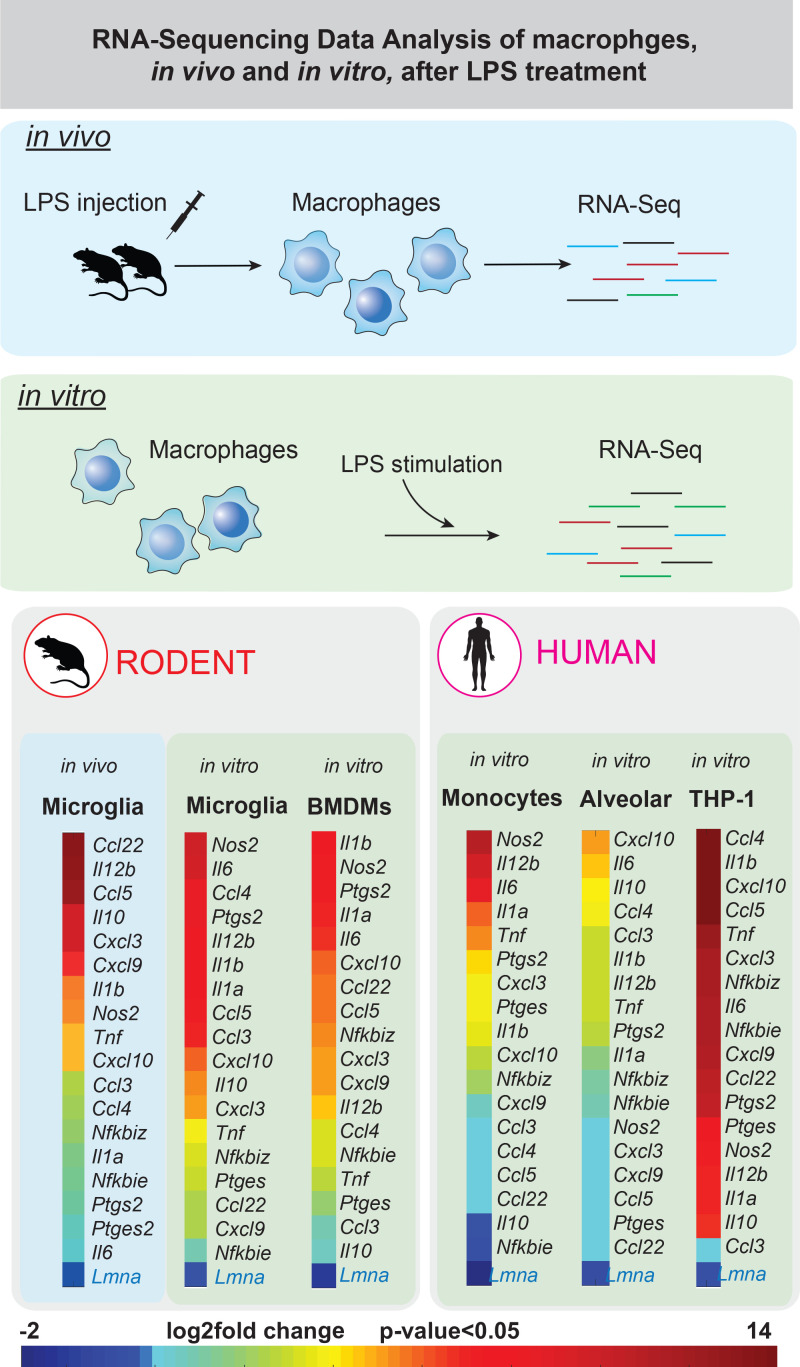
Pro-inflammatory macrophage activation results in a reversible lamin-A/C downregulation: Color coded map shows the mRNA expression levels of various pro-inflammatory genes and lamin-A/C in microglia of LPS-injected mice (5 mg/kg), in LPS-treated mouse microglia, bone marrow derived macrophages (BMDMs) and in LPS treated human monocyte and alveolar macrophages. Also shown are the expression levels in the LPS treated macrophage cell line THP-1 (human). Expression data were obtained from public repositories.^148–153^

Differentiation of rat and human macrophages has been shown to be accompanied by increased expression of lamin-A/C,^143^ which also increases during the differentiation of human peripheral blood monocytes into macrophages.^146^ Even though these studies have characterized the levels of lamin-A/C in different macrophage types, the central question which needs to be addressed is whether lamin-A/C expression has any potential role during macrophage inflammatory activation. A recent study from our lab suggests a potential and previously unknown role of lamin-A/C in regulating the pro-inflammatory response.^147^ Reanalyzing publicly available RNA-Seq data^148–153^ revealed that in several tissue-resident macrophages, both in mice and humans, upon LPS activation (both *in vitro* and *in vivo*), there is a significant decrease in the levels of *lamin-A/C* mRNA (Fig. 5). Concomitantly, the pro-inflammatory activation of BMDMs also results in a significant decrease in lamin-A/C protein levels.^147^ Previous studies have quantified the turnover rate of lamins in quiescent fibroblasts, revealing that around 10% of lamin-A/C is replaced within 24 h in the lamina meshwork with newly synthesized lamin-A/C proteins.^154^ Since a decrease in more than 50% is seen within 12–18 h of activation, lamin-A/C downregulation is an active process, which is due to lamin-A/C phosphorylation followed by its degradation.^147^ Mechanistically, lamin-A/C downregulation is necessary to drive pro-inflammatory gene expression, as inhibiting lamin-A/C degradation in BMDMs blocks pro-inflammatory gene expression (*IL-6* and *TNF-a*) and also pro-inflammatory cytokine secretion. Regarding tissue-resident macrophages, lamin A/C ablation in immune cells results in a selective depletion of lung alveolar macrophages and a heightened susceptibility to influenza infection.^155^ These alveolar macrophages also display DNA damage and p53-dependent senescence, hallmarks of inflammation and ageing, further confirming a potential role of nuclear lamina in macrophage function and inflammation. Finally, the overexpression of the lamin-A mutant progerin, a truncated version of the lamin-A protein,^156^ which does not properly integrate into the lamina and disrupts the nuclear lamina meshwork, leads to significant disfigurement of the nucleus.^156^ This induces endothelial cell dysfunction, characterized by increased inflammation and oxidative stress together with persistent DNA damage, increased expression of cell cycle arrest proteins, and cellular senescence, further providing proof of lamin-A/C as inflammatory regulator.^157^

Downregulation and degradation of the nuclear lamina are thus a mechano-regulated process and depend on a variety of physical parameters, including substrate stiffness.^158,159^ A recent study has revealed that lamin-A/C phosphorylation and turnover depend on the rigidity of the extracellular matrix. For example, mesenchymal stem cells (MSCs) grown on soft polymeric substrates show higher lamina degradation as compared to ones on stiff substrates.^158^ Even though the available evidence points toward a functional role of lamin-A/C during macrophage pro-inflammatory activation (Fig. 5), a detailed understanding of how the phosphorylation and subsequent degradation of lamin-A/C might drive inflammatory gene expression is lacking. The regulation could be at multiple levels starting from transcriptional regulation. Lamin-A/C has previously been shown to alter the spatial localization and activity of critical transcription factors like NF-κB^160^ and MRTF-A.^161,162^ One potential mechanism by which lamin-/C degradation could cause changes in a pro-inflammatory genomic program is by tuning the differential nuclear-to-cytoplasmic localization of these transcription factors. Similarly, there is a strong regulatory coupling between nuclear lamina and histone modifications (as discussed below in Sec. X). Thus, whether changes in lamin-A/C levels during M1 activation drive inflammatory histone modifications and epigenetic remodelling could be another interesting question to address. This includes probing major histone modifications like H3K4me3, a histone mark that is accumulated in the promoter region of pro-inflammatory genes upon LPS activation.^163^ At the same time, it is also absolutely crucial to explore the role of other nuclear envelope (NE) proteins in order to develop a more complete picture of the regulatory landscape by which macrophage activation is regulated by alterations of the NE, thus tuning the balance between pro-inflammatory and pro-healing macrophages.

## IX. STRUCTURAL REORGANIZATION OF INTRANUCLEAR SPACE UPON PROINFLAMMATORY ACTIVATION

Expression of the pro-inflammatory secretome requires access to the corresponding genes. It is worth mentioning that the changes in accessibility of chromatin and genes, as probed using Assay for Transposase-Accessible Chromatin using sequencing (ATAC-Seq), has shown that accessibility depends on the mechanical environment and mechanical status of cells. Uniaxial cyclic stretching of MSCs, for example, induces differentiation into osteoblasts by upregulating the chromatin accessibility of genes associated with the regulation of cell morphogenesis, cell–substrate adhesion, and ossification.^164^ Another study has shown that stiff ECM induces a tumorigenic phenotype through changes in nuclear morphology, chromatin state, and accessibility of chromatin.^165^ Cells cultured in stiff matrices displayed more accessible chromatin sites, which exhibited footprints of specific protein 1 transcription factor binding, and this transcription factor acts along with the histone deacetylases 3 and 8 to regulate the induction of stiffness-mediated tumorigenicity.^165^

During the past decade, genome-wide mapping methods have identified genomic regions that are in close contact with the nuclear lamina, termed lamina-associated domains (LADs).^166,167^ Transcriptionally inactive genes are generally positioned at the nuclear lamina and are part of these LADs;^135,168^ however, only ∼30% of LADs, as identified by sequencing, map to the nuclear periphery. LADs are of particular interest because most of the several thousands of genes in LADs are expressed at very low levels, thus suggesting a role in gene repression.^135,168^ Single-cell techniques like DamID, a method to capture contacts between DNA and a given protein of interest, have facilitated the identification of genomic regions in contact with the nuclear periphery and nuclear lamina.^169^ A few recent studies have shown that lamins differentially regulate distinct LADs at the nuclear periphery, which can in turn influence global 3D genome organization and gene expression.^44,170^ Gene activation inside LADs typically causes detachment of the entire transcription unit from the nuclear lamina, whereas inactivation of active genes can lead to increased nuclear lamina contacts.^131^ Lamin loss causes expansion or detachment of specific LADs in mouse embryonic stem cells (ESCs). The detached LADs disrupt interactions of both LADs and chromatin, thereby impacting genome organization and potentially genomic profiles.^44^ However, our understanding of LADs in macrophages is minimal. It is worth probing whether pro-inflammatory genes are part of these LADs and whether nuclear lamina degradation releases LADs in the interior of the nucleus so as to facilitate the expression of pro-inflammatory genes, which are otherwise silent and/or lowly expressed in resting macrophages.

Nuclear lamina-influenced changes in gene expression are also associated with changes in gene positioning. For example, knockdown of lamin A/C deregulates expression levels of genes; both KLK10 (Chr.19, LAD+) and MADH2 (Chr.18, LAD−) were significantly repressed, while BCL2L12 (Chr.19, LAD−) was de-repressed.^171^ These genes also reposition with respect to the nuclear envelope upon Lamin-A/C knockdown. One recent study showed the relocation of pro-inflammatory genes like TNF-α, a highly transcribed gene in LPS activated macrophages, within the nuclear space during macrophage activation.^172^ In contrast, the down-regulated genes did not change their position.^172^ A more detailed study involving several genes is required though to confirm that gene repositioning is necessary to regulate the pro-inflammatory protein expression. Whether and how nuclear lamina degradation is critical for this relocation is completely unknown.

Finally, it has been shown in several cell types that changes in the nuclear lamina and in lamin-A/C aid the repositioning of chromosomes due to altered interactions between chromosomes and the inner nuclear membrane. Three-dimensional DNA-immunoFISH revealed that the repositioning of chromosomal regions to the nuclear lamina is dependent on breakdown and reformation of the nuclear envelope during mitosis. During mitosis, chromosome movement correlates with reduced lamin association with the nuclear rim.^173^ This requires phosphorylation of lamin at sites analogous to those that open lamina network crosslinks in mitosis.^173^ Failure to remodel the lamina results in delayed meiotic entry, altered chromatin organization, and slowed chromosome movement.^173^ Nuclear lamina disruption in *Drosophila* S2 cells also leads to chromatin compaction and repositioning from the nuclear envelope.^174^ Additionally, the downregulation of lamin-A/C results in increased nuclear dynamics, thereby enabling relative displacement of chromosomes within the nucleus, leading to the formation of new chromosome surroundings and interactions. Even though similar processes are expected to regulate macrophage phenotypes, whether chromosome repositioning takes place during macrophage inflammatory activation and regulates their response remains unknown. Whether the phosphorylation induced degradation of the nuclear lamina during macrophage activation helps in chromosome repositioning is also not known and is worth future investigations to better characterize and fully understand the process of inflammatory activation.

## X. INFLAMMATORY EPIGENOME REGULATES GENE EXPRESSION PROFILES, BUT MECHANO-SENSITIVITY NEEDS INVESTIGATION

Epigenetics is the study of how cells control gene activity without changing the DNA sequence.^175^ Epigenetic changes/modifications are modifications to DNA that regulate whether genes are turned on or off and the set of modifications that regulate the expression of genes in a cell is termed the “epigenome.” The term, “epigenetics,” was first used to refer to the complex interactions between the genome and the environment that is involved in development and differentiation in higher organisms. Conrad Waddington coined the term “epigenetic landscape” defined by the molecular mechanisms that convert the genetic information into observable traits or phenotypes.^176^ Epigenetic modifications are either heritable chemical or physical changes in chromatin, and the main types of epigenetic modifications include histone modifications and DNA methylation. Gene expression is also influenced epigenetically by non-coding RNAs such as microRNA (miRNAs) and long non-coding RNA (lncRNAs).^177–180^ Epigenetic modifications steer the response of macrophages to external physical or biochemical stimuli and include the downstream secretion of pro-inflammatory or pro-healing factors. The secretome differs significantly between M1 and M2 activated macrophages,^12,181–185^ and the role of mechanical forces and physical factors is still being explored. The mechano-response of immune cells, however, might not necessarily be steered by the same epigenetic modifications as described so far for mesenchymal cells, in part due to their rather different cytoskeletal architecture, a possibility we will now explore further.

The first evidence of the link between LPS stimulation and epigenetic regulation in inflammatory genes dates back to 1999, as LPS stimulation induces the cytokine IL-12p40 production in murine macrophages by rapid and specific nucleosome translocation at the promoter region.^186^ A nucleosome is a section of DNA that is wrapped around a core of proteins. Each nucleosome is composed of a little less than two turns of DNA wrapped around a set of eight proteins called histones, which are known as a histone octamer. Each histone octamer is composed of two copies each of the histone proteins H2A, H2B, H3, and H4. With the advent of advanced sequencing techniques, changes in epigenetic modifications, mainly histone modifications and DNA methylation during macrophage inflammatory activation, have now been extensively probed.^9,182,187,188^ The development of chromatin immunoprecipitation assays in conjunction with advanced sequencing technologies has allowed researchers to probe different histone modifications and map the locations of specific proteins across the genome at high resolution during macrophage activation.^188,189^ LPS activation is a TLR4-dependent event and results in the acetylation and methylation of histones H3 and H4.^190^ Trimethylation of histone 3 lysine 4 (H3K4) is associated with active gene transcription, and trimethylation of H3K9, H3K27, and H3K79 is linked to silencing of gene expression during inflammation.^190^ As a major role of pro-inflammatory macrophages is to sterilize wound sites through the secretion of various cytokines, trimethylation of H3K4 on pro-inflammatory cytokine gene promoters must be induced in M1 macrophages in response to TLR stimulation.

Various histone modifications in response to alterations of physical properties of the cell microenvironment have recently been reported for mesenchymal cells. Acetylation of H3 on Lysine 9 (AcH3K9), for example, depends on the cell spreading area of fibroblasts.^100^ Similarly, stiff polymeric matrices lead to significantly higher levels of AcH3 but decreased levels of AcH4.^165^ Fibroblasts cultured in grooves (10 *μ*m in width and spacing) showed not only markedly increased global AcH3 marks, but also a significant increase in methylation (both di- and tri-methylation) of histone H3 at lysine 4 (H3K4me2 and H3K4me3, respectively) relative to flat surfaces.^191^ Increased levels of histone H3 acetylation have also been reported in mesenchymal stem cells cultured on elastic membranes patterned with parallel microgrooves (10 *μ*m wide).^192^ Importantly, other histone modifications are also sensitive to the physical properties of the cell microenvironment. Even in primary BMDMs, confining cells in a 3D environment induces trimethylation of histone 3 at residue K4 (H3K4me3).^193^ Several other histone modifications have been reported to be under the regulation of physical properties of the microenvironment, such as topography, lamina flow, substrate stiffness, and 3D collagen gels, and this has been reviewed extensively.^194^

**FIG. 6.**
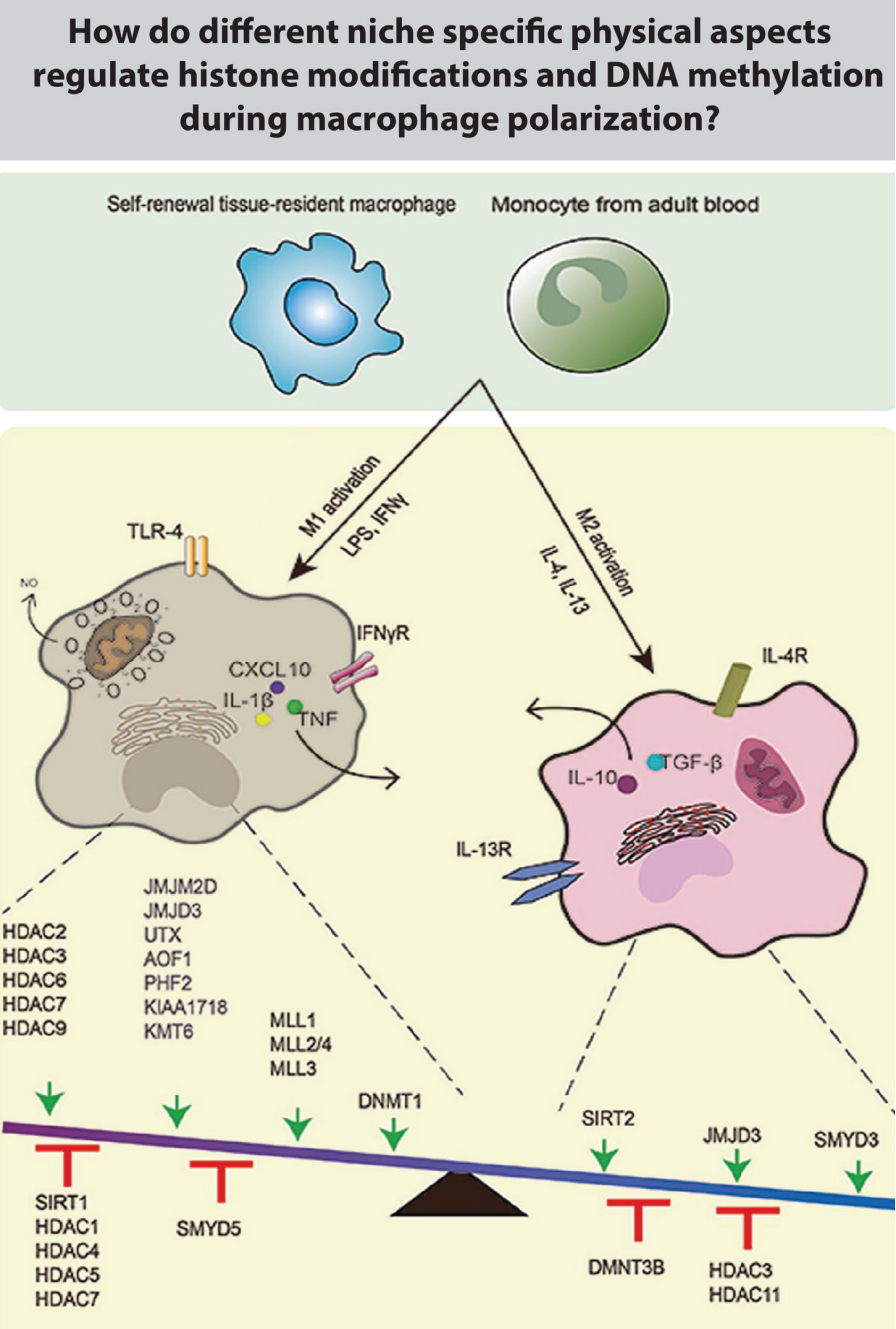
Mechano-regulation of enzymes involved in histone modifications and DNA methylations during macrophage polarization: Cartoon shows relevant epigenetic enzymes that regulate the macrophage phenotype as summarized by their influence. As shown in the balance model, the enzymes above the arrows have been shown to have activating effects, while those under the T-shaped support stand have repressive effects on M1/M2 activation. However, their regulatory dependence on different physical factors, which are known to exist in tissues, still needs to be probed. Cartoon adapted from.^190^ Reproduced with permission from Chen *et al.*, Cell Mol. Immunol. **17**, 36–49 (2020). Copyright 2020 Authors, licensed under a Creative Commons Attribution (CC BY) license.

In contrast, whether histone modifications induced during macrophage activation are cell niche dependent was not known until recently (Fig. 6). H3K36me2, a crucial histone modification to promote pro-inflammatory gene expression, is dependent on whether macrophages are free to spread or are spatially confined.^12^ Another central histone modification, AcH3 which is necessary for pro-inflammatory responses, has been shown to be dependent on the cell shape, being lower in elongated cells.^106^ The mechano-sensitivity of other modifications remains to be explored (Fig. 6). Even though it is well accepted that changes in cell spreading and confinement significantly alter nuclear architecture,^100,101^ whether the histone modifications are sensitive to these changes in the nuclear architecture has not been elucidated. Since chromatin is physically anchored to the nuclear lamina,^174,195–197^ it can be hypothesized that changes in the nuclear architecture and nuclear lamina could potentially drive differential histone modifications. In support of this, changes in the pattern of histone modifications in fibroblasts have been associated with mutations in A-type lamin, whereby heterochromatin markers, such as H3K9 trimethylation and heterochromatin-associated protein HP1γ, are reduced in cells with mutated lamin-A genes.^198–200^ In contrast, H4K20 trimethylation is increased in laminopathy fibroblasts, which have a lamin-A mutation.^201^ In addition, mutations in the lamin-A gene also result in a decreased level of H3K27 trimethylation on the inactive chromosome X.^201^ This shows that histone modifications are correlated and potentially regulated by lamin-A and nuclear lamina. Thus, we suggest a potential regulatory route by which changes in pro-inflammatory histone modifications during macrophage activation occur via mechanosensitive nuclear lamina and associated proteins.

Histone modifications are largely driven by histone methyltransferases (HMTs), histone acetyltransferases (HATs), and histone deacetylases (HDACs), which have been extensively reviewed elsewhere^51^ (Fig. 6). Even though HATs have been shown to be involved in initiating gene expression in macrophages during inflammation, only a limited number of reports have detailed to date how HATs catalyze the expression of specific M1 or M2 macrophage associated genes.^182^ In contrast, several HDACs, mainly HDAC3,^202,203^ are known to be involved in M1 activation and play a prominent role in the regulation of immunological pathways. In response to LPS, HDAC3-deficient macrophages are unable to induce the expression of several pro-inflammatory genes including *IL-6*.^12^ Insight into whether these histone remodeling enzymes are mechano-regulated, and whether their spatial localization and activity are driven by biophysical forces, has only recently been gained. HDAC3 nuclear translocation depends on macrophage spreading, being lower in spatially confined macrophages, establishing HDAC3 as a mechano-sensitive histone modification enzyme.^12^ Even though HDAC3 translocation in fibroblasts is dependent on actin polymerization,^100^ whether a similar mechanism exists in macrophages is still not known. p300 HAT enzymatic activity is also dependent on macrophage elongation. p300 is an important HAT associated with macrophage pro-inflammatory activation.^204^ LPS treated BMDMs, cultured on fibronectin coated micropatterned stripes, showed a lower enzymatic activity of p300 HAT,^106^ establishing p300 as a mechano-responsive histone remodeling enzyme. However, such data provide only preliminary evidence that the spatial localization and activity of HDACs and HATs, and potentially of other HMTs, are driven by a diverse set of mechanical and/or biochemical signals. Altogether, this provides first insight into the biophysical control of histone modification and chromatin remodeling enzymes and thereby how physical factors can regulate gene expression during macrophage activation.

**FIG. 7.**
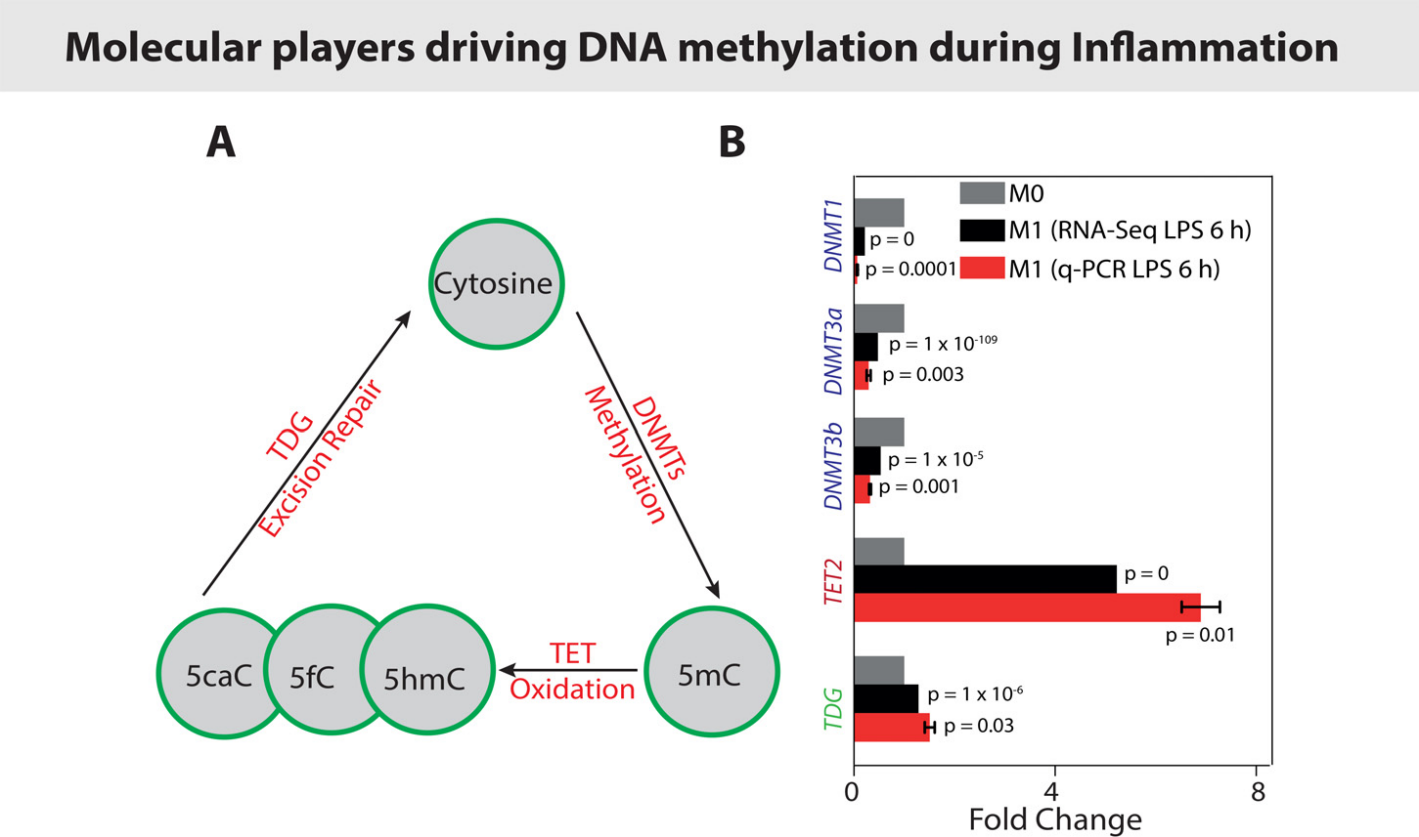
Changes in DNA Epigenetic modifications (DNA Methylation) during LPS induced pro-inflammatory macrophage activation: (a) depiction of cytosine methylation and demethylation processes. The different modified forms of cytosine (5mC, 5hmC, 5fC, and 5caC) along with the corresponding enzymes responsible for each modification are shown.^217^ (b) Bar graph shows the differential levels of various DNMTs, TETs, and TDG enzymes in M0 and M1 BMDMs as obtained from RNA-Seq and qPCR experiments, both performed after 6 h of LPS treatment.^217^ DNMTs: DNA methyltransferases; TET: ten-eleven translocation; TDG: thymine-DNA glycosylase. Data adapted from Ref. 217. Reproduced with permission from Jain *et al.*, Epigenetics **14**, 1183–1193 (2019). Copyright 2019 Taylor and Francis Group.^217^

DNA methylation, together with histone modifications, can also regulate gene expression.^205–207^ DNA methylation, and specifically methylation of the 5-carbon of cytosine (5 mC), is the most studied and among the most significant epigenetic modification^207,208^ [Fig. 7(a)]. DNA hypermethylation results in gene silencing by affecting the binding of methylation-sensitive DNA binding proteins and/or by further interacting with various histone modifications and co-repressors that alter DNA accessibility to transcriptional factors.^208,209^ DNA methylation is catalyzed by DNA methyltransferases (DNMTs) [Fig. 7(a)], including DNMT1, DNMT3a, and DNMT3b. DNMT1, which is responsible for DNA methylation maintenance, binds to methyl groups in hemimethylated DNA strands during DNA replication, whereas de novo DNMT3a and DNMT3b add methyl groups to CpG dinucleotides of unmethylated DNA.^206^ DNMT1 may also have a role in de novo DNA methylation.^210^ Recently, more attention has been given to 5-hydroxymethylcytosine (5hmC), which is an oxidation product of 5mC, and contrary to 5mC, the presence of 5hmC has generally been associated with increased gene expression^211–213^ [Fig. 7(a)]. The mammalian enzymes responsible for generating these modifications are the three ten-eleven translocation (TET) dioxygenases (TET1, TET2, and TET3) that utilize the co-factors α-ketoglutarate, reduced iron, and molecular oxygen to oxidize the methyl group (demethylation) at the 5 position of 5mC.^214–216^ Optical mapping techniques revealed that pro-inflammatory activation of BMDMs results in a significant decrease in the levels of DNMTs, i.e., DNMT1, DNMT3a, and DNMT3b with a concomitant increase in the levels of the TET2 enzyme [Fig. 7(b)].^217^

Whether DNA methylation is a mechano-regulated process remains elusive (Fig. 6). Only recently, it has been shown for various cell types, but not yet in macrophages, that DNA methylation depends on the biophysical properties of the cellular microenvironment.^194^ As cells engage in reciprocal interactions with their matrix, it is not surprising that DNA methylation is sensitive to ECM stiffness with global hypermethylation under stiff ECM conditions in mouse embryonic stem cells and embryonic fibroblasts compared with soft ECM.^218^ Stiff ECM enhances DNA methylation of both promoters and gene bodies, especially the 5′ promoter regions of pluripotent genes.^218^ The enhanced DNA methylation is functionally required for the loss of pluripotent gene expression in mESCs grown on stiff ECM, allowing them to differentiate along a specific lineage.^218^ Moreover, the altered DNA methylation is driven by ECM-regulated nuclear transport of DNA methyltransferase three-like (DNMT3L), which is promoted by a stiff ECM.^218^ Finally, using gastric cancer cells, it has been shown that the stiffness of the ECM reversibly regulates the DNA methylation of the promoter region of the mechanosensitive protein YAP.^219^ Similarly, seminal findings have been published showing that DNA methylation and DNA methyltransferases are also sensitive to shear stress.^220–222^ Both *in vitro* and *in vivo* blood flow models revealed that disturbed flow, as observed during atherosclerosis and characterized by low and oscillating shear stress, induces expression of DNMT1 and thereby regulates the genome-wide DNA methylation pattern. However, whether the tissue-dependent mechanical properties of microniches as discussed above drive macrophage homeostatic function and inflammation via DNA methylation remains unknown. As cells cannot only feel the stiffness of their microenvironment but also respond to the stretch-induced switching of the functional display of ECM fibers,^49^ future work is also needed to elucidate how changes of the mechanobiology of ECM alter the above-mentioned dependencies.

Finally, non-coding RNAs (miRNAs and lncRNAs) are important regulators of epigenetic modifications and, thus, gene expression, and their regulatory role during macrophage polarization has only recently been explored.^177–180^ Even though MicroRNA-503-5p inhibits stretch-induced osteogenic cell differentiation and bone formation^223^ and microRNA-103a functions as a mechanosensitive microRNA to inhibit bone formation through targeting Runx2,^224^ it is not yet known whether they play a mechano-regulated role in macrophage activation. While the mechano-sensitivity of epigenetic modifications and microRNA has been reviewed with a focus on endothelial and mesenchymal cells,^225–229^ our knowledge in the context of macrophages thus remains sparse.

## XI. PHYSIOLOGICAL IMPLICATIONS OF PHYSICAL CELL NICHE ALTERATIONS DURING INFLAMMATION AND AGE-ASSOCIATED INFLAMMATORY DISEASES

Physiological ageing is accompanied by a chronic, sub-clinical increase in pro-inflammatory cytokines (TNFα, IL-6) and reduced anti-inflammatory cytokines (IL-10) in the blood, termed inflammageing.^230,231^ This gradual progression with advancing age is a biomarker of ageing associated with an increased risk of several age-related diseases, including cardiovascular disease,^232^ sarcopenia,^233^ cancer,^234^ and dementia.^235^ Importantly, humans who reach very old age, i.e., centenarians, maintain a low inflammatory status with increased levels of anti-inflammatory cytokines, thus minimizing inflammageing.^236^ Understanding the causes of inflammageing enables better rational therapeutic strategies that would have broad health benefits, helping to deliver a healthy old age and prevent many age-related diseases and frailty.

While inflammageing is driven by many factors, a key contributor is the appearance of monocytes/macrophages that are in a state of low-level constitutive activation, resulting in the secretion of pro-inflammatory cytokines in the absence of infection.^237^ The differentiation of monocytes/macrophages to either a pro-inflammatory or more regulatory phenotypes is influenced by a variety of processes, including signals from cytokines but also in response to their physical environment as reviewed here. Research has shown that the direction of polarization can be influenced by ECM components as well as by culturing these cells on substrates of differing stiffnesses.^238,239^ Interestingly, the stiffness of the macrophage itself, again regulated by actin polymerization, also influences its phenotype. The mechanisms underlying the shift with age to pro-inflammatory phenotypes are poorly understood but appear to involve changes within cells and their response to microenvironmental biomechanical changes.

In a variety of cell types, there is increasing evidence that ageing is associated with changes in the mechanical properties of cells and strong correlations exist between age and cytoplasmic stiffness.^240^ Indeed, the mechanical stiffness of skin fibroblasts has been shown to correlate with biological age in humans.^17^ Ageing also influences the ability of cells to transduce biophysical changes into intracellular signals, altering the response of cells and tissues to mechanical forces.^241^ In the case of macrophages, the literature is focused on the response of these cells to their environment with age and little is known about age-related effects on macrophage stiffness. Recent studies have shown that culturing BMDMs from mice on substrates of increasing stiffness led to induction of a pro-inflammatory phenotype,^242^ confirming earlier reports of the ability of biomechanical forces to influence macrophage polarization.^243^ This earlier study also revealed that the mechanotransduction signal to achieve the inflammatory phenotype was mediated via transient receptor potential vanilloid 4 (TRPV4), a mechanosensitive ion channel.^243^ Thus, although the literature is sparse currently, there is support for the mechanical changes experienced by the macrophage in the aged environment contributing to its pro-inflammatory state and, thus, to inflammageing.

Other important contributors to inflammageing include senescent cells. These proliferatively quiescent cells are highly metabolically active, producing a complex pro-inflammatory secretome termed the senescence associated secretory phenotype (SASP).^244^ Senescent cells undergo profound morphological changes indicating an important role for mechanical signals in cell senescence.^245^ Senescent cells have increased vimentin, decreased actin, tubulin, and the focal adhesion protein paxillin.^245^ In the human progeria syndrome, Hutchinson–Gilford progeria syndrome increased cytoskeletal stiffness and RhoAGTPase activation in progeria cells was directly coupled with the morphological changes of cell senescence and induction of the pro-inflammatory response.^246^

As stated earlier, it is now widely recognized that most age-related diseases have a strong inflammatory component. The source of this inflammation is varied ranging from increased adiposity to reduced physical inactivity but includes the increased pro-inflammatory status of macrophages and cell senescence.^230,231^ That both cell and tissue mechanical properties change during disease is also now being appreciated with altered stiffness of cardiac muscle influencing the pro-inflammatory phenotype of infiltrating macrophages.^242^ In fibrotic diseases such as Idiopathic Pulmonary Fibrosis, matrix stiffening is evident and pathogenic, whereby α_6_-integrin is a matrix stiffness-regulated mechanosensitive molecule, which confers an invasive fibroblast phenotype.^187^ In neurodegenerative diseases such as Alzheimer's disease, a pathological feature is increased neuroinflammation, mediated through central nervous system based macrophages, the microglia. These cells, like their peripheral counterparts, have an activated, pro-inflammatory phenotype in Alzheimer's disease.^247^ Whether this is related to alterations in the stiffness of brain regions, for example, due to the presence of misfolded proteins such as amyloid, or simply a straight immune response to these proteins is yet to be determined.

## XII. CONCLUSIONS AND OUTLOOK

It is of uttermost importance to find cures for the many inflammatory diseases that are responsible for the majority of deaths and whose incidence increases with age. What has become evident is that an improved understanding of the role of mechanical forces in modulating the inflammatory status of cells such as macrophages and senescent stromal cells will deliver novel therapeutic options going forward. Creating new paradigms, which integrate biochemical, immunological, and mechanobiological factors, will produce significant new insights into age-related disease pathogenesis. Defining how to regenerate tissues affected by inflammatory pathologies, a major challenge in regenerative medicine, also requires mechanobiological knowledge. Since the demonstration that substrate stiffness correlates with mesenchymal stem cell differentiation fate,^24^ for example, many bioengineers focused on synthesizing new biomaterials that match tissue specific Young's moduli. Importantly, cells not only sense Young's modulus of their microenvironment: as they pull on the extracellular anchor points, they displace the adhesive ligands, which vice versa impacts integrin clustering and downstream mechanosensation,^23,25–28,41^ and at the same time stretch the ECM fibers, which can switch mechano-regulated molecular binding sites either on or off.^49,248–250^ As macrophages are major contributors to adverse inflammatory and fibrotic responses to implanted biomaterials,^251^ the development of immunomodulating biomaterials and of therapies to regenerate organs will require an improved understanding of how macrophage activation and polarization is steered by the physical properties of their niches, among all the other well described regulatory factors. Gaining a thorough understanding how physical properties of cell niches tune the pro-inflammatory response of macrophages, or if altered promote their pro-healing M2 phenotype, will thus be highly significant for many disciplines from cell biology to developmental biology. This knowledge will also impact medicine and translational approaches for novel therapies for age-related disease, as the efficacies of therapeutics are impacted by the complexity of the pathological cell niches. The identification of mechanosensitive targets in signaling cascades that regulate the pro-inflammatory or pro-healing phenotype can further be explored as new therapeutic targets.

## Data Availability

The data that support the findings of this study are available from the corresponding authors upon reasonable request.
